# Preparation and Performance Study of Novel Foam Vegetation Concrete

**DOI:** 10.3390/ma17246295

**Published:** 2024-12-23

**Authors:** Teng Zhang, Tianbin Li, Hua Xu, Mengyun Wang, Lingling Lu

**Affiliations:** State Key Laboratory of Geohazard Prevention and Geoenvironment Protection, Chengdu University of Technology, Chengdu 610059, China

**Keywords:** vegetation concrete, foaming agent, physical and mechanical properties, plant growth, slope protection

## Abstract

Vegetation concrete is one of the most widely used substrates in ecological slope protection, but its practical application often limits the growth and nutrient uptake of plant roots due to consolidation problems, which affects the effectiveness of slope protection. This paper proposed the use of a plant protein foaming agent as a porous modifier to create a porous, lightweight treatment for vegetation concrete. Physical performance tests, direct shear tests, plant growth tests, and scanning electron microscopy experiments were conducted to compare and analyze the physical, mechanical, microscopic characteristics, and phyto-capabilities of differently treated vegetation concrete. The results showed that the higher the foam content, the more significant the porous and lightweight properties of the vegetation concrete. When the foam volume was 50%, the porosity increased by 106.05% compared to the untreated sample, while the volume weight decreased by 20.53%. The shear strength, cohesion, and internal friction angle of vegetation concrete all showed a decreasing trend with increasing foaming agent content. *Festuca arundinacea* grew best under the 30% foaming agent treatment, with germinative energy, germinative percentage, plant height, root length, and underground biomass increasing by 6.31%, 13.22%, 8.57%, 18.71%, and 34.62%, respectively, compared to the untreated sample. The scanning electron microscope observation showed that the pore structure of vegetation concrete was optimized after foam incorporation. Adding plant protein foaming agents to modify the pore structure of vegetation concrete is appropriate, with an optimal foam volume ratio of 20–30%. This study provides new insights and references for slope ecological restoration engineering.

## 1. Introduction

Large-scale infrastructure and urbanization projects have resulted in numerous exposed slopes [1]. These slopes, when subjected to natural factors such as wind, sun exposure, and rainfall, are prone to geological disasters like collapses, landslides, and mudflows [2,3,4], posing severe threats to human life safety. Additionally, these events lead to a reduction in biodiversity, damage to landscape features, and create visual pollution due to their stark contrast with the surrounding natural scenery [5,6]. Traditional masonry measures, such as shotcrete, retaining walls, and frame beams, often sacrifice ecological functions and landscape structures without guaranteeing long-term protection [7,8,9]. With the global increase in environmental concerns, ecological protection of slopes has become increasingly important.

**Figure 1:**
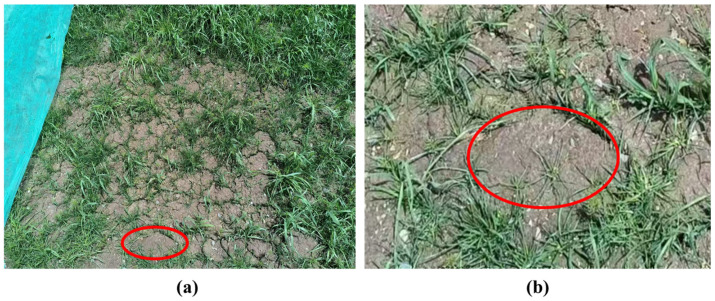
Vegetation concrete: (**a**) Vegetation concrete, (**b**) Consolidation of Vegetation concrete (Magnified view).

Vegetation concrete is a composite material that combines concrete with plant growth technologies [10]. It is made from planting soil, cement, aggregates, additives, fertilizers, water, and mixed green plant seeds [11]. This mixture is sprayed onto slopes using special equipment and is widely used for the comprehensive management and ecological restoration of exposed slopes, especially rocky ones. Vegetation concrete uses cement as a binder, and combined with common support materials such as anchors, dowels, and protective nets, it can stabilize the slope. Once the seeds germinate and grow into turf, the above-ground part of the vegetation can intercept rainfall, reduce splash erosion, and inhibit runoff. The roots form a dense reinforcement and anchorage system that further enhances slope stability [12,13]. However, in practical applications of slope ecological protection, it has been observed that the addition of cement can lead to consolidation of the vegetation concrete (Figure 1). This consolidation reduces the porosity and aeration of the vegetation concrete, adversely affecting root growth and nutrient uptake of the slope vegetation, ultimately hindering normal plant growth and compromising the effectiveness of ecological slope protection [14,15].

The consolidated vegetation concrete displays a reduction in porosity [16]. As the spatial environment for the extension of vegetation roots, vegetation concrete pores affect the growth of plant roots, determine the length and diameter of roots directly, and play an important role in slope protection [17,18]. Many scholars have improved the pore structure of vegetation concrete by adding plant fibers or residues. For example, Liu et al. [19] enhanced the pore structure of ecological slope protection substrates by adding organic materials such as rice husks, sawdust, corn, and distiller grains. Qiu et al. [20] increased the porosity by incorporating straw into the ecological slope protection substrate. These studies have improved the porosity of vegetation concrete; however, the plant fiber and plant residue can lead to discontinuous cracks in the vegetation concrete. Liu et al. [21] found that biochar could effectively increase the porosity of vegetation concrete. Currently, most methods to improve the porosity of vegetation concrete involve adding solid materials, which can increase the overall porosity. However, excessive amounts or uneven mixing can lead to agglomeration or the formation of cracks.

Lightweight foam concrete is a typical porous material characterized by low cost, lightweight, convenient construction, and green, low-carbon properties. It has been widely used in civil and industrial construction projects [22,23]. Foam concrete is made by incorporating foam from a foaming agent into concrete. The foaming agent is the primary substance that forms pores and can be divided into chemical and physical foaming agents [24,25]. Chemical foaming agents produce bubbles through chemical reactions, generating a large amount of gas but are challenging to control during gas production [26], and some foaming agents can produce harmful gases [27]. Physical foaming agents generate bubbles through mechanical action, mainly including rosin-based, synthetic surfactants, protein-based, and composite types [28], with more foam stability. Foaming agents are also used in soil improvement. For example, Vázquez et al. [29] and León et al. [30] used sugar foam as a soil amendment for planting vegetation, promoting crop growth; Chan et al. [31] found that Hypucem foam could improve soil physical properties, aiding healthy plant growth; Gao et al. [32] incorporated foaming agents into municipal sludge, inventing an artificial planting soil with a rich pore structure; Wang et al. [33] applied foaming agents in the formulation of plant growth soil, preparing improved soil that promoted vegetation growth. Currently, foaming agents are primarily used in the preparation of lightweight foam concrete and soil improvement, with fewer applications in the field of slope ecological protection.

To effectively reduce the consolidation of vegetation concrete and promote the growth of slope-protecting plants, this study attempts to apply a foaming agent as a modifier to vegetation concrete. Specifically, foam generated by the foaming agent is used to improve the pore structure of vegetation concrete, forming a lightweight, porous foamed vegetation concrete. Through experiments measuring volume weight and porosity, direct shear tests, plant growth tests, and SEM (Scanning Electron Microscope) analysis, the effects of incorporating foaming agents on the physical properties, mechanical properties, plant growth performance, and microstructure of vegetation concrete were systematically studied. Additionally, the mechanisms by which foaming agents influence the performance of vegetation concrete and slope ecological protection were also explored. This research provides new insights and references for slope ecological restoration projects.

## 2. Materials and Methods

### 2.1. Materials

This study was conducted based on the vegetation concrete developed by our research group [11,34]. The primary materials involved included planting soil, cement, aggregates, green additives, ecological magnetic slow-release fertilizers, plant fibers, and water. The planting soil is red-layer clay from the Sichuan Basin in China, sourced locally. It was cleaned of debris such as stones and dead branches, air-dried, and then sifted through a 2 mm sieve for use, with its basic physical properties shown in Table 1. The cement used is P·O 42.5R ordinary Portland cement produced in Sichuan. The aggregate consists of gravel and sand with a mass ratio of 2:1. A single-size grade is used, with the gravel particle size being less than 12 mm and the sand particle size being less than 4.75 mm. Green additives mainly include superabsorbent polymer (PAM), carboxymethylcellulose (CMC) as a binder, and plant residue as a pH regulator.

### 2.2. Experimental Design

**Figure 2:**
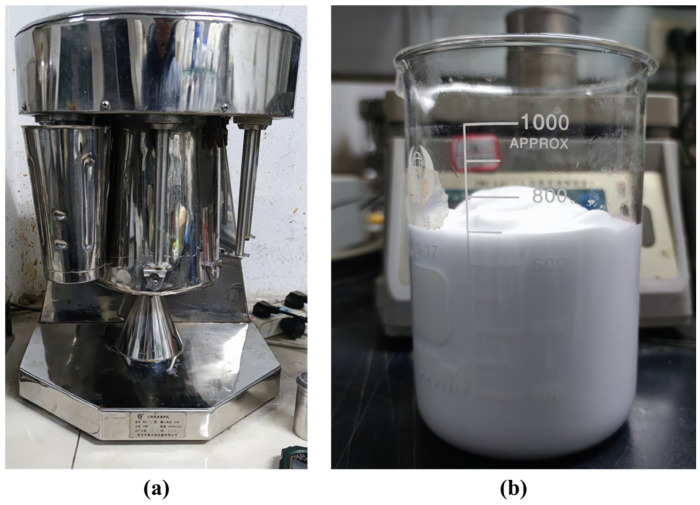
Foam made by high-speed mixer: (**a**) High-speed mixer, (**b**) Foam.

Protein foaming agents exhibit rapid foaming speed, uniform bubble size, and remarkably stable foam with superior foaming performance [35]. These agents can be classified into plant protein foaming agents and animal protein foaming agents [25]. The study used a plant protein foaming agent, which is characterized by its resistance to external factors such as temperature and pH, widespread production and applications, low cost, and environmental friendliness. The foaming agent was diluted with water at a ratio of 1:60 and then stirred using a high-speed mixer to produce foam, as shown in Figure 2. Six cases were established for the amount of foam added, corresponding to foam volume percentages of 0%, 10%, 20%, 30%, 40%, and 50% relative to the volume of vegetation concrete. The mix proportions of the vegetation concrete components are shown in Table 2.

### 2.3. Sample Preparation

**Figure 3:**
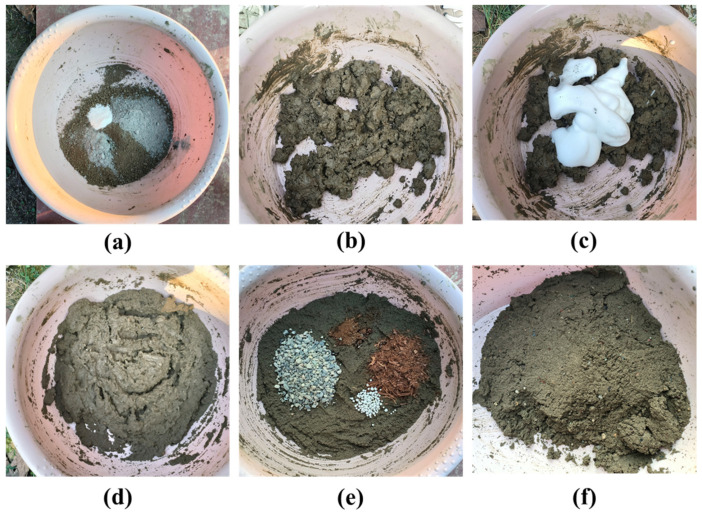
Sample preparation: (**a**) Add powdered components and stir; (**b**) Add water and stir; (**c**) Add foam. (**d**) Stirring, (**e**) Add larger granular components and stir; (**f**) Foam vegetation concrete.

According to the designed mix ratio, the components of vegetation concrete were weighed and mixed. First, powdered admixtures were thoroughly blended, followed by the addition of water to create a mixed slurry. The foaming agent was diluted with water and stirred to produce foam, which was then added to the mixed slurry according to the mix ratio and stirred until uniform. Subsequently, aggregates and plant fibers, which are larger-particle admixtures, were added and mixed evenly. The process of preparing the sample is shown in Figure 3. Specimens were prepared using a 60 cm^3^ cutting ring (diameter 61.8 mm × height 20 mm), wrapped in cling film immediately after preparation, and placed in a standard curing room (humidity above 95%, temperature 20 ± 2 °C) for curing. For the planting test, pots with a diameter of 15 cm and a height of 11.5 cm were used, with 50 *Festuca arundinacea* seeds sown in each pot. Watering was performed appropriately according to weather conditions to ensure consistent watering amounts per pot, and the germination and seedling growth of *Festuca arundinacea* seeds were monitored.

### 2.4. Experimental Methods

#### 2.4.1. Porosity

Three replicates were randomly selected from each treatment group, as described above. The porosity of vegetation concrete was measured using the mass method [36]. Porosity was calculated based on the apparent volume of the vegetation concrete specimen, the mass of the specimen when saturated in water, and the dry mass of the specimen, and the calculation formula is shown in Formula (1):

(1) $$P=\left(1-\frac{M_{1}-M_{0}}{V}\right)\times 100\%$$

In the formula, P is the porosity of the sample (%); V is the apparent volume of the specimen (cm^3^); M_0_ is the mass of the saturated specimen in water (g); M1 is the mass of the dried specimen (g).

#### 2.4.2. Volume Weight

Volume weight was measured using the drying and weighing method, and the calculation formula is shown in Formula (2):

(2) $$\rho =\frac{M-M^{\prime }}{V_{1}}$$

In the formula, ρ is the bulk weight of the test sample (g/cm^3^), M is the mass of the cutting ring plus the dried soil sample (g), $M^{\prime }$ is the mass of the cutting ring (g), and V_1_ is the volume of the cutting ring (cm^3^).

#### 2.4.3. Mechanical Properties

The direct shear test was conducted using a DZJ-4 multifunction direct shear apparatus, applying vertical pressures of 50 kPa, 100 kPa, 150 kPa, and 200 kPa, respectively. The shearing speed was controlled at 0.8 mm/min, and the test stopped when the displacement reached 10 mm. The peak shear stress from the test was used as the shear strength value. A graph of shear strength versus vertical pressure was plotted, with the angle of the line representing the internal friction angle and the intercept on the vertical axis representing the cohesion.

#### 2.4.4. Planting Performance

The germinative energy and germinative percentage of *Festuca arundinacea* were assessed at 7 and 10 days post-sowing, employing the method of dividing the total number of germinated seeds. Subsequently, plant height and underground biomass were measured 28 days after emergence. For each treatment, a random selection of 10 plants was made for measurement using a ruler. The underground portion was passed through a sieve and washed with water to determine the length of the primary root. Following this, the root was subjected to degreening in an oven at 105 °C for one hour before being dried at 80 °C until reaching constant mass; this dry weight represented the underground biomass.

#### 2.4.5. SEM

SEM testing was conducted using a FEI Nano SEM450 scanning electron microscope (FEI, Hillsboro, OR, USA) Specimens were cut into samples measuring 2 mm × 2 mm × 2 mm, and the surfaces of the specimens were coated with gold before observing the microstructure.

## 3. Results

### 3.1. Volume Weight and Porosity

**Figure 4:**
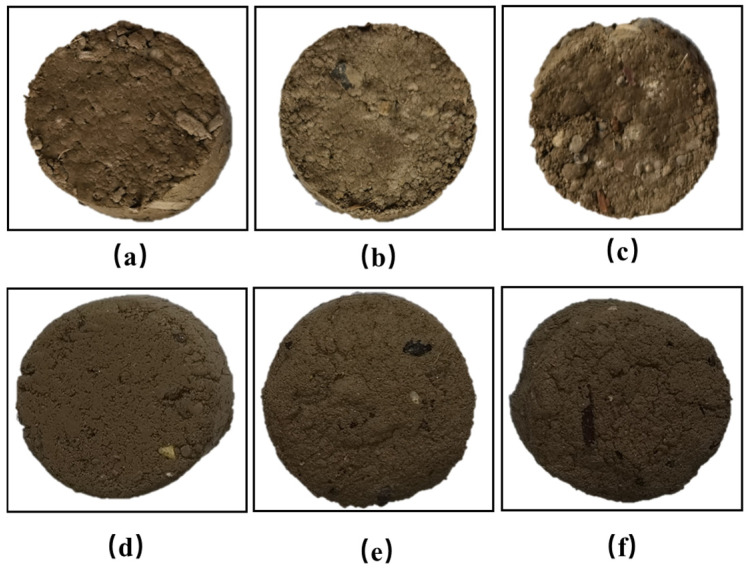
Vegetation concrete samples with different foam volumes: (**a**) 0%, (**b**) 10%, (**c**) 20%, (**d**) 30%, (**e**) 40%, (**f**) 50%.

Figure 4 shows the visual representation of vegetation concrete samples with different foam volumes after 28 days of curing. The samples with 0%, 10%, and 20% foam volume, shown in Figure 4a, Figure 4b, and Figure 4c, respectively, exhibit no significant changes in appearance. In contrast, the 30%, 40%, and 50% foamed specimens, shown in Figure 4d, Figure 4e, and Figure 4f, respectively, display a significant increase in surface porosity, with the 50% foamed specimen being the loosest. As the foam volume increases, the vegetation concrete becomes increasingly loose and porous. During sample preparation, it was found that adding foam caused the mixture to expand in volume and increase in porosity (Figure 3b,d). In addition, mixing became easier after the addition of foam.

**Figure 5:**
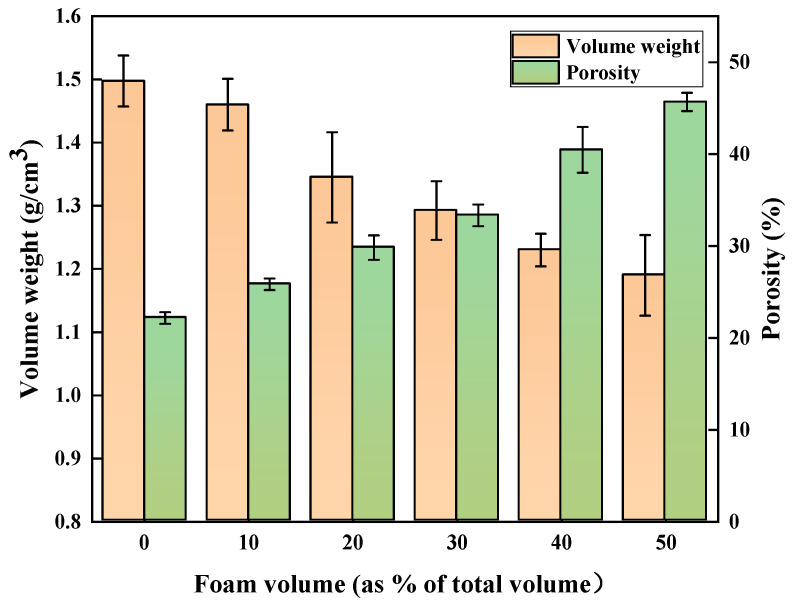
Change in volume weight and porosity of vegetation concrete with added foam volume.

The results of volume weight and porosity measured after 28 days of curing for vegetation concrete with different treatments are shown in Figure 5. It can be seen that with the increase in foam, the volume weight of vegetation concrete significantly decreased, while the porosity significantly increased. As a result, the characteristics of vegetation concrete, which are its porosity and lightweight nature, become increasingly evident. The volume weight of vegetation concrete with 50% foam volume was reduced by 20.53% compared to that without foam, while the porosity increased by 106.05%. When the foam volume was 40% and 50%, the difference in volume weight and porosity of vegetation concrete was only 0.04 g/cm^3^ and 5.21%, respectively, showing a tendency towards stability. The incorporation of foam cannot infinitely affect the volume weight and porosity of vegetation concrete; there is a threshold. Within a reasonable range, the porous and lightweight properties of vegetation concrete can reduce slope load, increase ventilation, improve drainage, and benefit slope stability, and lightweight vegetation concrete is more straightforward to construct and transport, helping to improve construction efficiency and reduce construction costs [37,38].

### 3.2. Shear Strength

**Figure 6:**
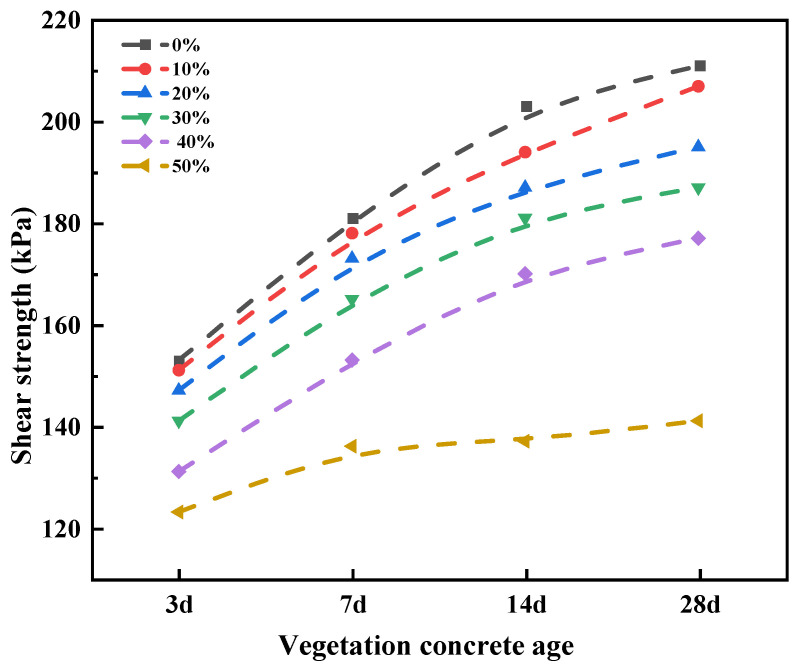
Shear strength of vegetation concrete at different ages.

Slope superficial layer failures are mostly shear failures [39]. The shear strength of vegetation concrete determines the shear stress and bearing capacity of slope vegetation concrete, which is one of the important mechanical properties affecting the stability of slope ecological protection [40]. The shear strength of vegetation concrete changes with age. Vegetation concrete with different treatments was selected for curing 3 d, 7 d, 14 d, and 28 d, and its shear strength under a vertical pressure of 200 kPa was measured. The results are shown in Figure 6.

Figure 6 shows the shear strength of vegetation concrete at different curing ages. It can be seen that with the extension of the curing age, the shear strength of vegetation concrete with different foam volumes continuously increases, with a rapid increase in the early stage, and the growth rate slows down from 14 days to 28 days. Using vegetation concrete with 30% foam volume as an example, the shear strength at 7 days increased by 24 kPa compared to 3 days, an increase of 17.02%. The shear strength at 14 days increased by 16 kPa compared to 7 days, an increase of 9.70%. The shear strength at 28 days increased by 6 kPa compared to 14 days, an increase of 3.31%. Within the studied range of foam volume, as the foam volume increases, the shear strength of vegetation concrete at the same curing age shows a downward trend, with the decreasing rate gradually increasing. At 7 days, when the foam volume ratio is 10%, 20%, 30%, 40%, and 50%, the shear strength of vegetation concrete is 178 kPa, 173 kPa, 165 kPa, 153 kPa, and 136 kPa, respectively, which is a decrease of 1.66%, 4.42%, 8.84%, 15.47%, and 24.86% compared to vegetation concrete without foam additives.

**Figure 7:**
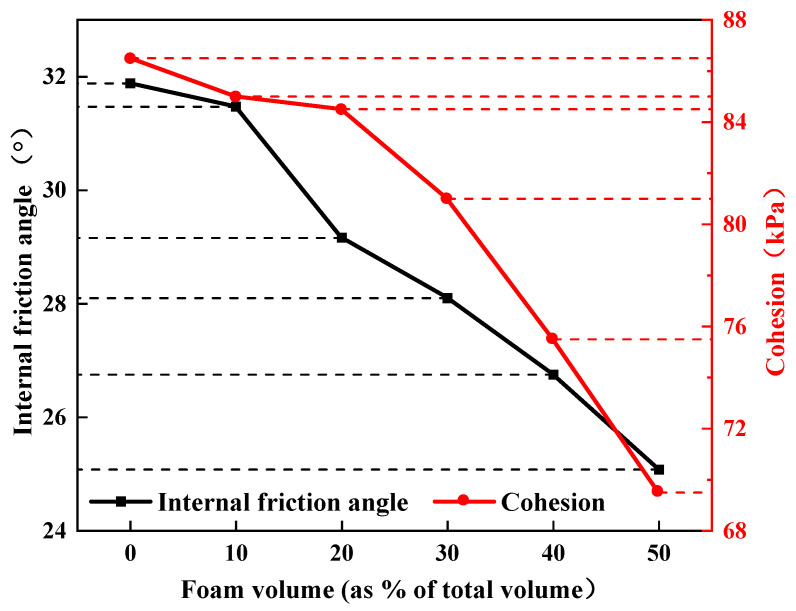
Influence of foam volume on cohesion and angle of internal friction of vegetated concrete.

Draw the relationship curve between the shear strength of vegetation concrete at 28 days under different treatments and vertical pressure and obtain its cohesion and internal friction angle, as shown in Figure 7. As the foam volume increases, the cohesion and internal friction angle of vegetation concrete decrease significantly, with the maximum decrease in cohesion being 17 kPa and the maximum decrease in internal friction angle being 6.8°.

### 3.3. Planting Properties of Festuca Arundinacea

**Figure 8:**
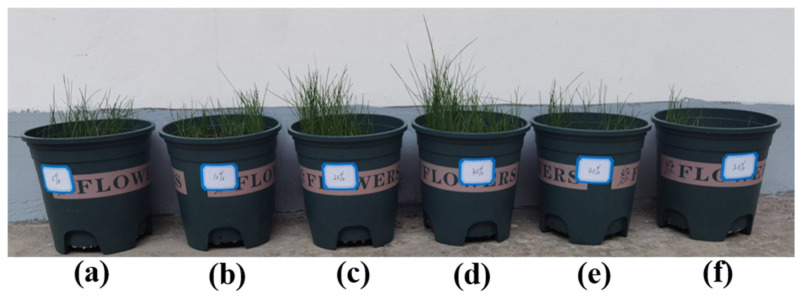
The growth conditions of *Festuca arundinacea* on vegetation concrete with different treatments: (**a**) 0%, (**b**) 10%, (**c**) 20%, (**d**) 30%, (**e**) 40%, (**f**) 50%.

*Festuca arundinacea* is a common grass species in the slope areas of Southwest China, characterized by its perennial growth habit, well-developed root system, rapid growth rate, and strong adaptability. The growth conditions of *Festuca arundinacea* on vegetation concrete with different treatments are shown in Figure 8.

**Figure 9:**
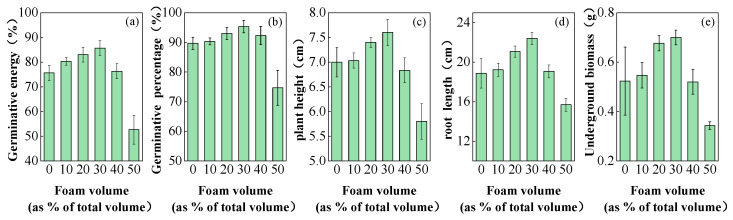
Influence of foam volume on the biomass of *Festuca arundinacea*.

Figure 9 illustrates the effect of foaming agent dosage on the biomass of *Festuca arundinacea*. The figure demonstrates that germinative energy (Figure 9a), germinative percentage (Figure 9b), plant height (Figure 9c), root length (Figure 9d), and underground biomass (Figure 9e) all show a trend of increasing before decreasing as the foaming volumes increase. At foam volumes of 10%, 20%, and 30%, all growth indicators for *Festuca arundinacea* were higher than those of the blank control group. At a foam volume of 40%, the plant height was lower than that of the blank control group, while other indicators were higher or equal to the blank control group. At a foam volume of 50%, all growth indicators for *Festuca arundinacea* were lower than those of the blank control group. In the 30% foam treatment, *Festuca arundinacea* grew best, with germinative energy, germinative percentage, plant height, root length, and underground biomass increasing by 6.31%, 13.22%, 8.57%, 18.71%, and 34.62%, respectively, compared to the treatment without foaming agent.

### 3.4. Microstructure

**Figure 10:**
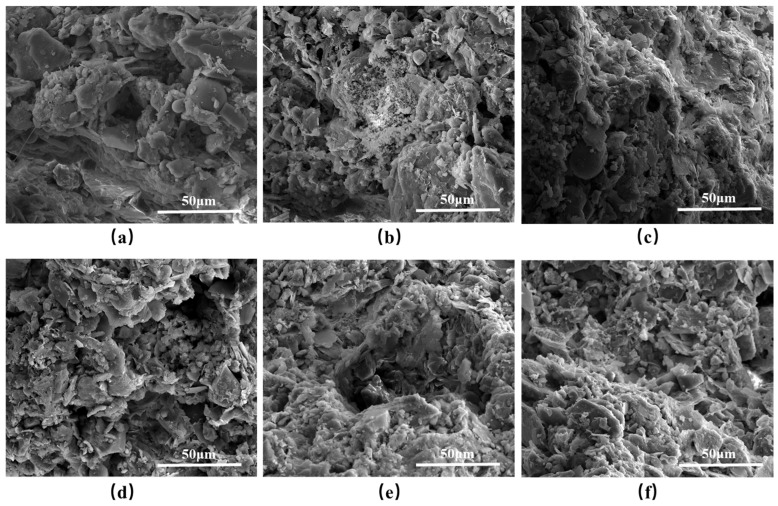
SEM images of vegetation concrete samples at ×2000 magnification: (**a**) 0%, (**b**) 10%, (**c**) 20%, (**d**) 30%, (**e**) 40%, (**f**) 50%.

**Figure 11:**
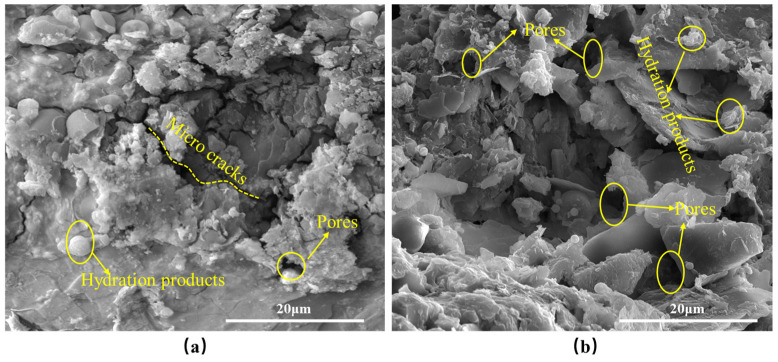
SEM images of vegetation concrete with 0% and 50% foam volume at ×5000 magnification: (**a**) 0%, (**b**) 50%.

To further investigate the changes in the internal structure of vegetation concrete upon the addition of a foaming agent, scanning electron microscopy (SEM) tests were conducted on samples that had been cured for 28 days. The findings are presented in Figure 10 and Figure 11. Specifically, Figure 10 displays the SEM images of vegetation concrete with varying foaming agent concentrations, magnified to 2000×. Additionally, Figure 11 illustrates the SEM images of vegetation concrete with 0% and 50% foam volume content, magnified to 5000×.

From Figure 10, it is evident that the microstructures of the vegetation concrete vary based on the foaming agent dosage. The unfoamed sample (Figure 10a) exhibits a dense structure with larger particles and fewer pores. With the introduction of the foaming agent, both the number of pores and the fineness of the particles in the vegetation concrete increase. As the foaming agent content rises, the pore structure of the vegetation concrete becomes finer, and the distribution and proportion of pores become more uniform. Figure 11a illustrates that without any foam, the vegetation concrete has fewer internal pores, and some microcracks are present. However, when the foam volume content reaches 50% (Figure 11b), there is a significant increase in the number of pores, and the internal structure undergoes substantial changes.

## 4. Discussion

### 4.1. The Effect of Foaming Agents on the Physical Properties and Plant Growth Performance of Vegetation Concrete

The incorporation of foam not only creates pores within the vegetation concrete, thereby increasing its porosity but also reduces the volume of the mixture per unit of space, which consequently lowers the volume weight of the vegetation concrete. Consequently, the resulting vegetation concrete exhibits a lightweight and porous characteristic. Volume weight and porosity are important parameters for evaluating soil structure [41], representing the physical properties of the soil. High volume weight and low porosity indicate compacted soil with poor structural integrity. Conversely, a low volume weight and high porosity indicate good soil structure [42]. A reduction in volume weight and an increase in porosity generally signify improved vegetation concrete structure. This is corroborated by Figure 10 and Figure 11, which illustrate that the foaming agent significantly enhances the pore structure within the vegetation concrete, leading to a more homogeneous distribution of pores throughout the cement-soil matrix.

The germinative energy, germinative percentage, plant height, root length, and underground biomass of *Festuca arundinacea* exhibit a trend of initial increase followed by a decrease with the increasing dosage of foaming agents, reaching a maximum at 30% foam volume. Beyond this point, the growth-promoting effect on *Festuca arundinacea* diminishes, demonstrating that while the addition of foam promotes growth, more is not necessarily better. The findings of Wang et al. [43] suggest that soil pores are of significant importance in the context of soil water retention and fertility maintenance. The capacity of soil pores to retain water, nutrients, and air directly impacts the soil’s ability to retain water, nutrients, and air, which in turn affects soil fertility and the ecological status of vegetation [44,45]. An appropriate foam dosage leads to a favorable pore structure in vegetation concrete, which is conducive to water retention and air circulation, providing the necessary conditions of moisture and oxygen for the germination of *Festuca arundinacea* seeds and the growth of their root systems. However, exceeding the optimal dosage leads to increased porosity, weakening the water and nutrient retention capabilities of vegetation concrete and inhibiting seed germination and seedling growth.

Vegetation plays a crucial role in slope ecological protection. It intercepts rainfall and slows down water flow through its aboveground parts, protecting the soil surface from wind and rain erosion. Additionally, vegetation stabilizes the soil through its roots, strengthens soil structure, and improves soil chemical properties [46]. For *Festuca arundinacea*, high germination percentage and energy are beneficial for protecting slope surfaces at an early stage. An increase in plant height can significantly reduce surface runoff and soil erosion [47]. An increase in underground biomass enlarges the contact area between roots and soil, enhancing the friction between them. This enhances the interaction between roots and soil, increasing the shear strength of the root-soil composite [48,49]. Overall, an appropriate foam dosage is conducive to improving slope stability.

### 4.2. The Impact of Foaming Agents on the Strength of Vegetation Concrete

After treatment with cement, the planting soil in vegetation concrete transitions from contact bonding to cementation bonding [50], thereby forming a cement-soil framework structure composed of soil particles, cement particles, hydration products, and crystalline products [51]. This enhances the structural strength of the soil. However, the introduction of foam increases inner porosity, reducing the cementation effect and affecting the mechanical properties of vegetation concrete itself. This observation is consistent with the study by Ma et al. [52]. They noted that the strength of soil-based foamed concrete primarily depends on the pore structure of the matrix and the bonding interface. When the foam content increases, it enlarges the inner pores, significantly reducing the compressive strength of concrete.

The cohesion of vegetation concrete primarily depends on the crystallization and cementation between particles. As foam volume increases, the cement-soil particle framework fills more pores, reducing the cementation force and making the overall structure prone to damage, leading to decreased cohesion. The internal friction angle is related to the sliding of soil particles and their rearrangement. With increasing foam volume, more pores replace the connections between particles, reducing the direct contact area between particles. Consequently, the internal friction angle also decreases.

Vegetation concrete must meet specific shear strength requirements to serve its purpose in slope protection. Huang et al. [53] conducted research indicating that to prevent shear slippage between vegetation concrete and the slope surface, the cohesion required to maintain self-stability of vegetation concrete on a 70° slope should be no less than 15 kPa, and the internal friction angle is generally maintained at around 30°. In this study, all sample cohesions were greater than 15 kPa, except for samples with 40% and 50% foam volume, where the rest had internal friction angles ranging between 28° and 32°, meeting slope protection requirements.

### 4.3. Suitable Foam Volumes

The introduction of foam into vegetation concrete enhances its lightweight and porous characteristics, but it also leads to a decrease in strength, resulting in both positive and negative effects. The positive effects of foam include increased porosity and reduced volume weight, while the negative effect is a reduction in strength. By carefully selecting the appropriate foam volume, it is possible to maximize the benefits of the foaming agent while keeping the drawbacks within acceptable limits. A foam volume ratio of 20% to 30% is considered optimal for achieving an excellent pore structure and suitable porosity in vegetation concrete, which promotes seed germination and plant growth while ensuring that shear strength and other performance indicators meet slope protection standards.

Additionally, when the incorporation volume ratio of foam is between 20% and 30%, the reduction in volume weight ranges from 10.2% to 13.7%. This translates to a savings of approximately 205 to 276 kg/m^3^ in raw materials. Consequently, there is a decrease in the use of sand, gravel aggregates, and cement, which contributes positively to environmental protection and promotes the efficient use of global resources.

## 5. Conclusions

This paper demonstrates the use of plant protein foaming agents to create lightweight vegetation concrete, enhance its pore structure, reduce consolidation, and promote the germination and growth of *Festuca arundinacea* seeds. A lightweight porous vegetation concrete suitable for ecological slope protection was developed, leading to the following conclusions:

(1) Incorporating foam made from plant protein foaming agents into vegetation concrete as a modifier effectively increased the porosity and reduced the volume weight. The higher the dosage was, the more significant the lightweight and porous properties of the vegetation concrete became. When the foam volume was 50%, the porosity increased by 106.05% compared to the untreated sample, while the volume weight decreased by 20.53%. The germinative energy, germinative percentage, plant height, root length, and underground biomass of *Festuca arundinacea* exhibited a trend of initial increase followed by a decrease. *Festuca arundinacea* grew best in the 30% foaming agent treatment, with all phytoindicators higher than in the no foaming agent treatment.
(2) As the foam dosage increased, the shear strength of vegetation concrete gradually decreased. Additionally, both cohesion and internal friction angles exhibited a downward trend. When the foam content is ≤30%, it can meet the requirements for slope protection. However, at the same foam dosage, the shear strength of vegetation concrete increased progressively with the curing age. Scanning electron microscopy observations revealed that after incorporating plant protein foaming agent foam, the pore structure of vegetation concrete became finer, and the proportion of pore structures became more uniform, significantly improving the pore structure. The optimum volume ratio for incorporation of plant protein foaming agent foam is 20–30%, which ensures that the mechanical properties meet the requirements for slope stability while maximally improving the physical properties and plant growth environment of vegetation concrete.
(3) This research provides a new technical approach to ecological slope protection. Optimized lightweight porous vegetation concrete can reduce slope loading, improve soil aeration and drainage, reduce consolidation, and promote the growth of slope vegetation, which helps to improve slope stability and reduce construction costs. Our study focused primarily on examining how a plant protein-based composite foaming agent influences the performance characteristics of vegetation concrete; validation was conducted mainly in controlled laboratory settings. To advance the field, future research should investigate the adaptability of this newly developed foamed vegetation concrete to different environmental conditions and assess its long-term durability.

## Figures and Tables

**Table 1:** Basic physical properties of soil samples.

Specific Gravity *G*s	Dry Density (g/cm^3^)	Void Ratio	Liquid Limit (%)	Plastic Limit (%)	Permeability Coefficient *k* (cm/s)
2.68	1.59	0.82	49	23	2.7 × 10^−6^

**Table 2:** Vegetation concrete proportioning.

Soil (kg/m^3^)	Aggregate (kg/m^3^)	Cement (kg/m^3^)	Green Additive (kg/m^3^)			Ecological Magnetic Slow-Release Fertilizer (kg/m^3^)	Plant Fibers (kg/m^3^)	Water (kg/m^3^)
			PAM	CMC	pH Regulator			
1500	400	70	4.5	5.5	8	15	15	500

## Data Availability

Date are contained within the article.

## References

[1] Bao X., Liao W., Dong Z., Wang S., Tang W. (2017). Development of Vegetation-Pervious Concrete in Grid Beam System for Soil Slope Protection. Materials.

[2] Xu Y., Su C., Huang Z., Yang C., Yang Y. (2022). Research on the Protection of Expansive Soil Slopes under Heavy Rainfall by Anchor-Reinforced Vegetation Systems. Geom. Geomembr..

[3] Huang Z., Peng Z., Jiao W., Liu Y., Xu Y., Ma S. (2024). Field Study on Vegetation Eco-Protection Technology for Red Sandstone Fill Slope against Water Damage. Case Stud. Const. Mater..

[4] Mickovski S.B., Gonzalez-Ollauri A., Sorolla A., Löchner A., Emmanuel R. (2024). A Case History of Co-Design and Co-Deployment of a Nature-Based Solution (NbS) against Erosion and Slope Instability. Ecol. Eng..

[5] Li Y.H., Zhao J.Z., Zhang W.H., Guo R., Nan G.J., Li Y.C. (2021). Research Status and Development Trend of Ecological Restoration Technology for High and Steep Rock Slope in Open-Pit Mine. J. Hebei GEO Univ..

[6] Tomassi A., Falegnami A., Meleo L., Romano E. (2024). The GreenSCENT Competence Frameworks. The European Green Deal in Education.

[7] Wang J.-P., Bai M.-H., Tan Y.-R., Ge S.-Q., Gao X.-G., Dadda A., Shen J.-Y., Zhang J. (2024). Effect of Vegetation on Unsaturated Soil Hydraulic and the Slope Stability under Rainfall. Rhizosphere.

[8] Bai Y., Liu J., Xiao H., Song Z., Ma K., Deng Y. (2023). Soil Stabilization Using Synthetic Polymer for Soil Slope Ecological Protection. Eng. Geol..

[9] Yao D., Qian G.P., Yao J.L., Liu J.W., Yu X.L. (2020). Polymer Curing Agent in Ecological Protection Design Weak Rock Slope Engineering Application. J. Perform. Constr. Facil..

[10] Liu C., Xia Y., Chen J., Huang K., Wang J., Wang C., Huang Z., Wang X., Rao C., Shi M. (2023). Research and Application Progress of Vegetation Porous Concrete. Materials.

[11] Xu H., Li T.B., Chen J.N., Liu C.N., Zhou X.H., Xia L. (2017). Characteristics and Applications of Ecological Soil Substrate for Rocky Slope Vegetation in Cold and High-Altitude Areas. Sci. Total Environ..

[12] Yang L.X., Li S.C., Sun H.L., Ye F.F., Liu W., Luo S. (2011). Polyacrylamide Molecular Formulation Effects on Erosion Control of Disturbed Soil on Steep Rocky Slopes. Can. J. Soil Sci..

[13] Alam S., Manzur T., Borquist E., Williams J., Matthews J.C. (2021). In-situ Assessment of Soil-Root Bonding Strength to Aid in Preventing Soil Erosion. Soil Till. Res..

[14] Chang J., Im G.C., Cho G.C. (2016). Introduction of Microbial Biopolymers in Soil Treatment for Future Environmentally-Friendly and Sustainable Geotechnical Engineering. Sustainability.

[15] Chang M., Lee A.T., Tran S., Lee Y.M., Kwon J., Im G.C., Cho G.C. (2020). Review on Biopolymer-Based Soil Treatment (BPST) Technology in Geotechnical Engineering Practices. Transp. Geotech..

[16] Finch H.J.S., Samuel A.M., Lane G.P.F., Finch H.J.S., Samuel A.M., Lane G.P.F. (2014). Soils and Soil Management. Lockhart & Wiseman’s Crop Husbandry Including Grassland.

[17] Zhang J., Ran Y.G., Ma D.H., Chen L., Wu Y.B., Huang P. (2024). Study on the Growth Dynamics of Cynodon dactylon Roots and Their Impact on Soil Pore Evolution. Acta Pedol. Sin..

[18] Tian L., Pang Z.M., Quan H.Z., Wang J., Li Z.X., Wang H.J. (2016). Study on the Physical Properties and Vegetation Adaptability of Plant-Growing Porous Concrete. J. Chin. Ceram. Soc..

[19] Liu D.X., Li S.L., Xu W.N., Cheng Z.L. (2012). Selection Tests for Type and Ratio of Organic Matter in Vegetation Concrete. Adv. Sci. Technol. Water Resour..

[20] Qiu C., Han X.Z., Chen X., Lu X., Yan J., Feng Y., Gan J., Zou W., Liu G. (2021). Effects of Organic Amendment Depths on Black Soil Pore Structure Using CT Scanning Technology. Trans. Chin. Soc. Agric. Eng..

[21] Liu D., Gao X., Xu Y., Yang Y., Chen J., Ding Y., Xia D., Xiao H., Xu W. (2021). Influence of Biochar Addition Amount on Physicochemical Properties of Vegetation Concrete and Biomass of Cynodon dactylon. J. Basic Sci. Eng..

[22] Guo M., He Y., Zhi X. (2024). Experimental Research on Dynamic Mechanical Properties of High-Density Foamed Concrete. Materials.

[23] Shang X., Qu N., Li J. (2022). Development and Functional Characteristics of Novel Foam Concrete. Constr. Build. Mater..

[24] Yan Z., He Y. (2015). High-Performance Foam Concrete Insulation Products: Practical Technology.

[25] Gu Y., Wang Y., Wang X. (2013). Research Progress of Foam Concrete in Different Processes. J. Build. Mater..

[26] Liu X., Jiao S., Wang Z. (2017). A Review on Foaming Agents and Foam Concrete. Value Eng..

[27] Rong H., Zhang J., Zhang L., Zhang Y., Xu R., Liu Z. (2020). Preparation and Performance of Bio-Based Foaming Agents for Foam Concrete. Bull. Chin. Ceram. Soc..

[28] Yan Z., He Y. (2021). Foam Concrete Foaming Agent Production and Application Technology.

[29] Vázquez E., Teutscherová N., Almorox J., Navas M., Espejo R., Benito M. (2017). Seasonal Variation of Microbial Activity as Affected by Tillage Practice and Sugar Beet Foam Amendment under Mediterranean Climate. Appl. Soil Ecol..

[30] León P., Espejo R., Gómez-Paccard C., Hontoria C., Mariscal I., Renella G., Benito M. (2017). No-Tillage and Sugar Beet Foam Amendment Enhanced Microbial Activity of Degraded Acidic Soils in South West Spain. Appl. Soil Ecol..

[31] Chan C.-L., Joyce D.C. (2007). Effects of Urea Formaldehyde Foam Soil Amendment on Growth and Response to Transient Water Deficit Stress of Potted Flindersia schottiana Saplings. Sci. Horticult..

[32] Gao H., Zhang B., Zhou K., Cheng F. (2021). Preparation Method for Municipal Sludge-Based Artificial Planting Soil. Chinese Patent.

[33] Wang Y., Dong B., Xing F., Hong S., Fang G., Zhang Y. (2022). A Method for Preparing Landscape Planting Soil Particles and Methods for Using the Same. Chinese Patent.

[34] (2023). Technical Guidelines for Ecological Protection of Highway Slopes Using Mesh Anchorage and Spraying Vegetation Concrete.

[35] Zhao Y., Wu Z. (2016). Effect of Foaming Agent Content on Properties of Foamed Cement Banking. J. Pingdingshan Univ..

[36] Kim H.H., Park C.G. (2016). Plant Growth and Water Purification of Porous Vegetation Concrete Formed of Blast Furnace Slag, Natural Jute Fiber, and Styrene Butadiene Latex. Sustainability.

[37] Qu H.L., Wang C.X., Huang X.L., Ding Y., Huang X. (2020). Seismic Performance of Substrate for Vegetation Concrete from Large-Scale Shaking Table Test. Shock Vib..

[38] Liu D., Liu D., Gao J., Yang Y., Ding Y., Guo C., Zhang X., Xia Z., Xu W. (2022). Influence of Addition of Two Typical Activated Carbons on Fertility Properties and Mechanical Strength of Vegetation Concrete under Freeze-Thaw Conditions. Sci. Total Environ..

[39] Wang Y., Liu X., Zhang Z., Ma D., Cui Y. (2015). Experimental Research on Influence of Root Content on Strength of Undisturbed and Remolded Grassroots-Reinforced Soil. China J. Geotech. Eng..

[40] Jia S. (2011). Reinforcement Mechanism and Mechanical Properties of Lime-Cement Soil. Master’s Thesis.

[41] Yu B., Zhao L.P., Gao J.L., Wang X.C., Li Y., Liu X.Y., Zhang X.Y., Wang H.J., Li S.Y., Wang X.M. (2012). Study on Response and Mechanism of Soil Porosity to Freezing in High-Yield Farmland in Songliao Plain. J. Soil Water Conserv..

[42] Yang W., Lan H., Li M., Meng C. (2021). Predicting Bulk Density and Porosity of Soil Using Image Processing and Support Vector Regression. Trans. Chin. Soc. Agric. Eng..

[43] Wang Y., Su Z., Zhou M. (2020). Characteristics and Influence of Topsoil Porosity in the Northern Agro-Pastoral Ecotone. Pratacultural Sci..

[44] Li H., Fan S., Zhang G., Zhang S., Zhou Z. (2010). Characteristics of Soil Water Holding and Soil Porosity under Different Tree Species after Conversion of Cropland to Forest in the Loess Hilly Region. Bull. Soil Water Conserv..

[45] Pei Z., Wu Y., Wang Q., Zhong Z., Ren M., Wei C., Lu J., Wang W. (2016). Correlations between Soil Porosity Related Parameters and Other Soil Parameters in Songnen Plain, Northeastern China. Res. Soil Water Conserv..

[46] Zuazo V.H.D., Pleguezuelo C.R.R., Lichtfouse E., Navarrete M., Debaeke P., Véronique S., Alberola C. (2009). Soil Erosion and Runoff Prevention by Plant Covers: A Review. Sustainable Agriculture.

[47] Lann T., Bao H., Lan H., Zheng H., Yan C., Peng J. (2024). Hydro-mechanical Effects of Vegetation on Slope Stability: A Review. Sci. Total Environ..

[48] Xu H., Wang X.Y., Liu C.N., Chen J.N., Zhang C. (2021). A 3D Root System Morphological and Mechanical Model Based on L-Systems and Its Application to Estimate the Shear Strength of Root-Soil Composites. Soil Tillage Res..

[49] Xu H., Yuan H.L., Wang X.Y., Wang D., Chen J.X., Rong C.Q. (2022). Influences of Morphology and Hierarchy of Roots on Mechanical Characteristics of Root-Soil Composites. Chin. J. Geotech. Eng..

[50] Wang Q., Chen H.E., Cai K.Y. (2003). Quantitative Evaluation of Microstructure Characteristics of Cement-Soil. Rock Soil Mech..

[51] Xiong J., Li Y., Hu S., Liao C., Zhou M., Wang J., Luo S. (2024). Influence of Different Parent Soils on the Microstructure and Strength of Cemented Soil. Bull. Chin. Ceram. Soc..

[52] Ma C., Chen B. (2015). Properties of a Foamed Concrete with Soil as Filler. Constr. Build. Mater..

[53] Huang X. (2011). Effect of Herb Roots on Shearing Strength in Vegetation-Growing Concrete Matrix. Master’s Thesis.

